# Skyrmion Dynamics in a Double-Disk Geometry under an Electric Current

**DOI:** 10.3390/nano12183086

**Published:** 2022-09-06

**Authors:** Sebastián Castillo-Sepúlveda, Javier A. Vélez, Rosa M. Corona, Vagson L. Carvalho-Santos, David Laroze, Dora Altbir

**Affiliations:** 1 Grupo de Investigación en Física Aplicada, Facultad de Ingeniería, Universidad Autónoma de Chile, Avda. Pedro de Valdivia 425, Providencia 7500912, Chile; 2 Departamento de Polímeros y Materiales Avanzados: Física, Química y Tecnología, Universidad del País Vasco, UPV/EHU, Paseo M. Lardizabal, 3, 20018 San Sebastián, Spain; 3 Donostia International Physics Center, 20018 San Sebastián, Spain; 4 Departamento de Física, CEDENNA, Universidad de Santiago de Chile, Avda. Víctor Jara 3493, Estación Central, Santiago 9170022, Chile; 5 Departamento de Física, Universidade Federal de Viçosa, Avenida Peter Henry Rolfs s/n, Viçosa 36570-000, MG, Brazil; 6 Instituto de Alta Investigación, Universidad de Tarapacá, Casilla 7D, Arica 1000000, Chile

**Keywords:** magnetic skyrmions, spin torque nano-oscillators, dynamics, micromagnetism

## Abstract

In this work, we present an analysis of skyrmion dynamics considering Dzyaloshinskii–Moriya interactions in an STNO device with a double-disk geometry. Three regimes were observed as a function of geometric parameters and the electric current density: (i) the skyrmion is annihilating at the system’s border; (ii) the skyrmion moves in a non-circular trajectory alternating its position between the two disks, and (iii) the skyrmion only rotates inside a one-disk subsystem. For the annihilation state, we found that the transient time decays within a stretched exponential law as a function of the electric current. Our results show a 2D state diagram that can guide new experimental work in order to obtain these specific behaviors for new applications based on skyrmion dynamics.

## 1. Introduction

Magnetic skyrmions are solitonic magnetic textures presenting topological protection, which ensures strong stability for the particle-like textures [1,2,3]. This property makes skyrmions good candidates for several applications, such as multi-state memories and logic or radio-frequency devices, to mention a few [2,3,4,5,6]. Therefore, analyzing the skyrmion static and dynamical properties is a highlighted topic in nanomagnetism. Experimental works have revealed that skyrmions can be stabilized on thin ferromagnetic films by dipolar interactions, Dzyaloshinskii–Moriya interactions (DMIs), curvature effects [7], as well as frustrated exchange interactions [8]. The skyrmion’s properties (such as chirality or size) depend on the kind of interaction stabilizing it, the magnetic parameters of the material, and temperature [9,10]. For instance, if DMI is present to stabilize the skyrmion, its size is determined by the DMI, anisotropy, and exchange stiffness constant strengths [11].

Some examples of materials where the DMI is responsible for the appearance of magnetic skyrmions are MnSi and Fe${}_{1-x}$Co${}_{x}$Si, with typical whirl areas in the range of ∼10−100 nm [4] and ∼1 nm [2], respectively. In the case of MnSi systems, a helical magnetic order is observed below a transition temperature in the absence of magnetic fields. Therefore, in such systems, skyrmions can be stabilized through external magnetic fields and are only visible in a tiny part of the magnetic state diagram [12,13]. Nevertheless, if one considers skyrmions as metastable states, various skyrmion lattices can emerge at low temperatures, increasing the range of parameters where this topological structure can be observed [14].

From the perspective of the dynamics, it was shown that when a skyrmion is nucleated in a nanodisk, and a time-dependent stimulus is applied, it rotates around its axis with a certain eigenfrequency that depends on the material parameters, temperature, and frequency of the external driving force [15,16,17,18]. Indeed, when a spin-polarized current is injected into the magnetic material that hosts a skyrmion, a torque is created, allowing it to bifurcate its motion from a stationary to a non-stationary phase [18]. This current-induced skyrmion motion allows the use of the skyrmion dynamical properties as a spin torque nano-oscillator (STNO), with a strong potential for magnetic information manipulation. In this regard, Guo et al. [19] designed a ferromagnet/spacer/ferromagnet/heavy metal STNO model system to investigate the dynamics of skyrmions by adjusting several geometrical and material parameters. The results evidence the possibility of avoiding the Magnus-force-induced annihilation of skyrmions. Additionally, Jin et al. [20] proposed an STNO system that confines the skyrmion to the geometry-induced potential of an annular groove in a magnetic nanodot. In this case, the precession frequency of the skyrmion is more than six times higher than the frequency when it moves in a nanodot without the annular groove. Finally, Gobel et al. [21] studied the motion of a single skyrmion in a nanodisk in the absence of external currents, showing that it moves toward the center of the nanodisk in a spiral trajectory. In this case, the skyrmion motion suffers acceleration and deceleration while traveling toward the disk’s center.

It is well established that the geometry of the nanoparticle plays an essential role in skyrmion stabilization [22,23,24] and motion [25,26,27]. Moreover, border effects influence the skyrmion dynamics since boundary conditions are crucial for determining the magnetization properties of nanostructures hosting skyrmions. In this context, recently, Castillo-Sepúlveda [28] analyzed the trajectory of a skyrmion moving on an asymmetric disk, showing that the radius and frequency of the skyrmion precession motion depend on the disk shape and electric current. In this case, two main regimes for the skyrmion motion were obtained due to the competition between the restoration torque promoted by the disk borders and the spin-transfer torque. Below a critical parameter representing the asymmetry value, the skyrmion precession converges to stagnation states. Above this threshold, the skyrmion rotates in the nanodisk on a trajectory given by asymmetric circumferences.

Based on the above, through micromagnetic simulations, we analyze skyrmion dynamics under the action of an electric current when it is hosted in a system formed by two interconnected disks. When the electric current is zero, the skyrmion reaches its equilibrium position, which depends on the center-to-center distance of the nanodisks. When a non-null electric current is applied, our results evidence the emergence of three dynamical regimes for the skyrmion motion, depending on the degree of the disk’s connection and the external electric current: (i) the skyrmion is annihilated at the system’s border; (ii) the skyrmion performs a global oscillation, moving in a non-circular trajectory and alternating its core position between the two disks, and (iii) the skyrmion performs a rotation motion in a local trajectory, just inside a one-disk subsystem. These results allow us to obtain a complete state diagram as a function of the electric current and the system’s geometrical parameters.

The manuscript is organized as follows: in Section 2, we describe the adopted theoretical model. Section 3 presents the obtained results and discussions. Finally, the conclusions are given in Section 4.

## 2. Theoretical Model

**Figure 1:**
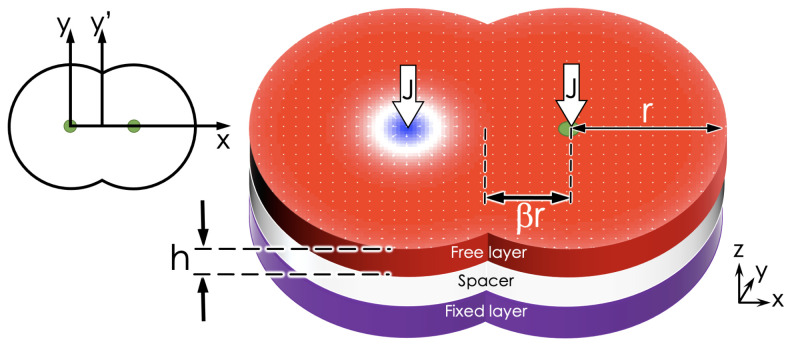
Schematic representation of an STNO system with a double disk geometry. A spin-polarized current is injected through each dot axis.

A usual STNO device consists of three stacked layers. The top and bottom layers are ferromagnetic and separated by a third layer, consisting of a thin non-magnetic slab. The bottom layer, also called the fixed layer, keeps its magnetization fixed and perpendicular to the interfaces. In contrast, the top layer’s magnetization is free to orient itself in some direction that depends on the conditions of the system. For our study, we consider a double-interconnected disk geometry, as shown in Figure 1. Such geometry is characterized by three parameters: the center-to-center distance w=2rβ, the radius *r*, and the thickness *h*. In the center of both disks, there are electrodes that allow the injection of a polarized current through each disk. Additionally, the free layer is a chiral ferromagnet with a high anisotropy induced by a non-magnetic metal, allowing the stabilization of a skyrmion.

The dynamics of the reduced magnetization $\vec{m}=\vec{M}/M_{s}$ are given by the Landau–Lifshit–Gilbert–Slonczewski (LLGS) equation

(1) $$\frac{d\vec{m}}{dt}=-\gamma \vec{m}\times {\vec{H}}_{\mathrm{eff}}+\alpha \left(\vec{m}\times \frac{d\vec{m}}{dt}\right)+{\vec{T}}_{\mathrm{STT}}\;,$$

where γ is the gyro-magnetic ratio, ${\vec{H}}_{\mathrm{eff}}=-\left(1/\mu_{0}M_{s}\right)\delta E/\delta \vec{m}$ is the effective field, and *E* is the energy density, given by the sum of the exchange ($E_{x}$), dipolar ($E_{d}$), anisotropy ($E_{a}$), and DMI ($E_{d}$) contributions; that is, $E=E_{x}+E_{m}+E_{a}+E_{d}$. Additionally, α is a dimensionless dissipation parameter, and ${\vec{T}}_{\mathrm{STT}}$ is the spin transfer torque term due to out-of-plane spin-polarized current, given by

(2) $${\vec{T}}_{\mathrm{STT}}=\frac{J}{\xi }\vec{m}\times \left({\vec{m}}_{P}\times \vec{m}\right)-\zeta \frac{J}{\xi }\vec{m}\times {\vec{m}}_{P}\;.$$

Here, $\xi =2\mu_{0}\left|e\right|hM_{s}/\left(\gamma \hbar P\right)$, where $\mu_{0}$ is the vacuum permeability, *e* is the fundamental charge, h is the height of the free layer, $M_{s}$ is the saturation magnetization, *ℏ* is the reduced Planck constant, *P* is the polarization. Also, ${\vec{m}}_{P}$ is the polarization vector, and ζ is the amplitude of the out-of-plane torque relative to the in-plane torque.

To numerically solve the LLGS equation, we use the Mumax3 code [29], which is a fast, widely known code that allows good control of the geometry. Likewise, its results show good agreement with the experiments. The physical parameters used in the simulations of the free layer are a stiffness constant A=15 pJ/m, a saturation magnetization $M_{s}=5.8$ MA/m, and a damping constant α=0.01. These parameters allow to define systems where at least one skyrmion can be nucleated, as shown in Ref. [30]. Moreover, the non-magnetic-metal induces a DMI of magnitude D=3.0 mJ/m${}^{2}$ in the free layer and a uniaxial anisotropy given by K=0.8 MJ/m${}^{3}$. The magnetization of the fixed layer is given by ${\vec{m}}_{P}=-\hat{z}$, and the non-magnetic material is described by ζ=0.1 and P=0.3. In our calculations, we consider that parameter *J* ranges from $J=1\cdot 10^{10}$ to $2.5\cdot 10^{12}$ A/m${}^{2}$. Finally, the initial conditions in all simulations consist of a skyrmion nucleated in the neighborhood of the left electrode (see Figure 1).

From considering the skyrmion as a rigid structure, where the skyrmion does not deform during its motion, its trajectory can be determined by the skyrmion core’s position as a function of time. This rigid body approximation allows us to use the Thiele formalism [31] for the skyrmion position, given by

(3) $$\vec{G}\times \vec{V}+D\cdot \vec{V}+\vec{F}=0\;,$$

where $\vec{G}=G_{z}\vec{z}$ is the gyrocoupling vector, D is the dissipation dyadic tensor, and $\vec{V}=\dot{\vec{R}}$. Here, $\vec{F}={\vec{F}}_{b}+{\vec{F}}_{\mathrm{STT}}$, with ${\vec{F}}_{b}$ being the restoration-like force that the system’s border exerts on the skyrmion, and ${\vec{F}}_{\mathrm{STT}}$ is the spin transfer torque (STT) force. The origin of ${\vec{F}}_{\mathrm{STT}}$ lies in the out-plane electric current injected into the system, whose magnitude is proportional to the current density *J*. Both forces, ${\vec{F}}_{b}$ and ${\vec{F}}_{\mathrm{STT}}$, depend on $\vec{R}$.

### 2.1. Some Insight into Symmetric and Asymmetric Circular STNO-Like Devices

To have some insight into the behavior of a skyrmion propagating under the action of an electric current in the interconnected disk system, we can make use of some properties of the effective forces originating from the system’s borders and qualitatively discuss the motion of the position of a particle-like texture as that given by Equation (3). Indeed, due to the azimuthal symmetry of circular STNO-likes devices, we can state that ${\vec{F}}_{b}=-F_{b}^{\rho }\hat{\rho }$, while ${\vec{F}}_{\mathrm{STT}}=F_{\mathrm{STT}}^{\rho }\hat{\rho }+F_{\mathrm{STT}}^{\theta }\hat{\theta }$, where $\hat{\rho }$ and $\hat{\theta }$ are the radial and tangential unitary vectors of cylindrical coordinates, respectively.

One can notice that the radial force exerted by the disk borders works as a restoring force, pointing to the center of the nanodisk, and depends on the distance *r* between the skyrmion core and disk center. On the other hand, the radial component of the effective force is associated with the STT points from the disk center to its border. Therefore, for a certain threshold radius $R_{\mathrm{eq}}$, we have $F_{\mathrm{STT}}^{\rho }=F_{b}^{\rho }$. Nevertheless, the tangential component of the STT force is unbalanced, creating a precessional motion of the skyrmion, which describes an orbit with radius $R_{eq}$ around the disk center. The frequency and radius of the orbit depend on *J*. If *J* is high enough, the skyrmion is annihilated at the disk border [18].

Contrarily to their circular counterparts, asymmetric circular STNO-likes devices present a tangential component to the restoration force [28]; that is, ${\vec{F}}_{b}=-F_{b}^{\rho }\hat{\rho }-F_{b}^{\theta }\hat{\theta }$. Therefore, from the competition between the restoring and STT forces, two main dynamical regimes emerge when a skyrmion displaces in such STNO-like devices. The first consists of a precessional motion, where the skyrmion rotates in a non-circular orbit around a stationary point determined by the balance between the normal and the border components of the effective forces. The second regime occurs in stagnation points, where the skyrmion stops its motion in a stationary position (fixed point), where ${\vec{F}}_{b}+{\vec{F}}_{\mathrm{STT}}=0$; that is, both total components of the forces are null. Again, when *J* is above a certain threshold and the skyrmion reaches the disk borders, border-states appear that finally end up annihilating the skyrmion [28].

### 2.2. Two-Disk STNO-Like Device

Due to the absence of azimuthal symmetry, the system considered in this work exhibits qualitatively similar behavior to that of asymmetric circular STNO-like devices. Nevertheless, the interconnected disks present two main stationary positions near the centers of each subsystem and two electrodes that allow the application of electric currents. Therefore, the skyrmion motion in the interconnected disk presents differences compared to the system with just one disk. First, because the system has no azimuthal symmetry, the restoring force should have components pointing normally and tangentially to the borders. Then, the skyrmion equilibrium position at zero current is not above the electrode and depends on β, as will be discussed after in this text. Secondly, when a spin-polarized current is injected into each electrode, the skyrmion moves following different trajectories depending on the system’s condition. Indeed, in this case, the force $\vec{F}$ has three contributions: one restoration force ${\vec{F}}_{b}$ and two STT forces ${\vec{F}}_{\mathrm{STTL}}$ and ${\vec{F}}_{\mathrm{STTR}}$, where ${\vec{F}}_{\mathrm{STTL}}$ (${\vec{F}}_{\mathrm{STTR}}$) is the STT force due the electric current in the left (right) disk. Since the functional form of the ${\vec{F}}_{b}$ in the two-disk geometry is more complex than that for circular STNO devices, we can expect the emergence of different dynamical regimes for its motion. These regimes should appear due to the competition between the effective forces acting on the skyrmion and are directly related to the degree of interconnection between the disks and the applied external currents.

To better analyze all the possible dynamical regimes of skyrmions in the two-disk system, in the next section, we present the results obtained from micromagnetic simulations exploring the effect of both the degree of the disk’s connection (β) and the external electric current density (*J*).

## 3. Results and Discussion

To better understand our results, we organize this section into two subsections. The first subsection briefly analyzes the case in which there is no external current. The second subsection presents the skyrmion dynamical properties when we inject an electric current into the two electrodes. In both cases, the effect of the disk’s connection is analyzed, considering that β varies from 0.2 to 0.9. Additionally, we adopt values of *J* ranging from 0.01 to 2.5 A/μm${}^{2}$. The time of the dynamics is 0.5μs, which is a long enough time for the skyrmion trajectories to reach the permanent state. In all simulations, we use the initial condition described in Section 2.

### 3.1. Equilibrium States without Current

**Figure 2:**
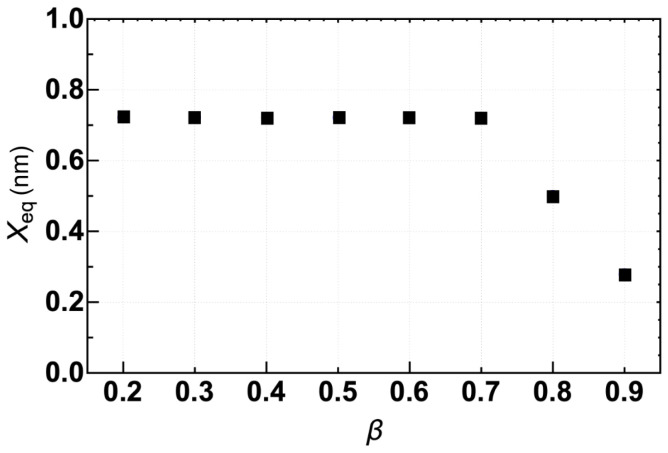
*x*-component of equilibrium state, ${\vec{R}}_{eq}$, as a function of β at J=0.

The first step to properly describing the skyrmion behavior on the interconnected disk is to find the equilibrium position of the skyrmion in the absence of an electric current. Starting our simulations with a skyrmion nucleated in the left disk, we observe that after a transient, the skyrmion’s core reaches the equilibrium position ${\vec{R}}_{eq}$. The stable point is defined by $\vec{F}\left({\vec{R}}_{eq}\right)=\vec{0}$. Our results show that the two stable points are very close to the center of each disk. For instance, taking the center of the left disk as the origin, the analysis of the *x*-component $X_{eq}$ shown in Figure 2 reveals that the equilibrium position of the skyrmion for β≲0.7 is almost constant and takes the value of 0.7 nm. Nevertheless, for β>0.7, $X_{eq}$ decreases linearly with β. Therefore, it is possible to conclude that only for large β values, β≈ 0.9, the system behaves as quasi-isolated subsystems. In this case, the radial component of the effective force pulls the skyrmion to the disk’s center while the tangential component is almost null.

We also call attention to the fact that there is an unstable point in the center of the whole structure. In this case, the skyrmion would stay at rest if we adopted this point as the initial condition of the simulations.

### 3.2. Effect of the Current: State Diagram

**Figure 3:**
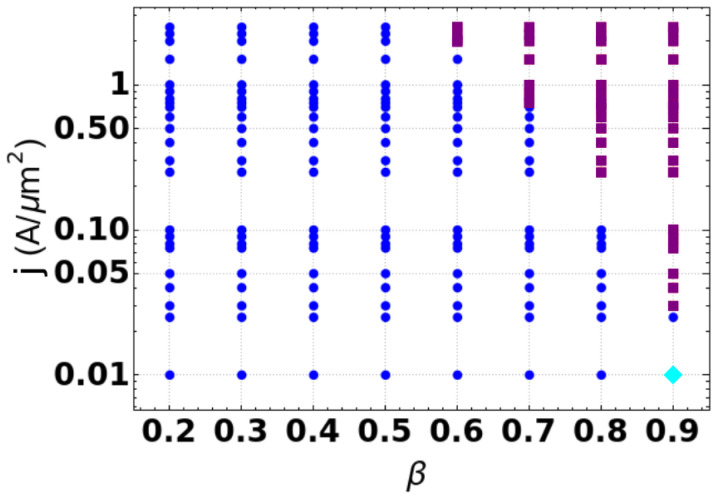
Diagram of the skyrmion’s dynamical behaviors as a function of β and *J*. The (blue) dots represent the state of a global oscillatory regime. The (purple) squares display the state of skyrmion annihilation. The (cyan) diamond depicts the state of a local oscillation.

After analyzing the equilibrium position of the skyrmion as a function of β, we perform a set of numerical calculations for different values of *J* and examine the trajectories tracking the skyrmion’s core. Figure 3 depicts a state diagram (SD) of the regimes that the skyrmion exhibits during its motion as a function of β and *J*. Three different dynamical regimes are shown. In the first regime, represented by purple squares in the SD, the skyrmion annihilates at the system’s border during its motion. One can observe that this regime appears for large values of electric current and β. Indeed higher currents increase the radius of the skyrmion orbit. Then, due to the small area of the region connecting the discs for large β, the skyrmion is pulled through the disk’s border, annihilating there. The other two regimes consist of oscillatory states. One of them is global (blue dots in Figure 3), which means that the skyrmion moves along the system, with its trajectory covering part of both disks. In this case, there is enough space in the region connecting the disks, and the skyrmion performs its orbit without annihilating at the system’s border. The second observed oscillatory motion is local (cyan diamonds in SD), and the skyrmion motion is localized in just one disk. This last state is observed for small values of electric current and large values of β. Indeed, as stated before, for larger values of β, the disks should behave as quasi-insulated systems, and the STT effective force is not large enough to compete with ${\vec{F}}_{b}$ originating in the interconnecting region. Therefore, the skyrmion keeps oscillating in only one disk.

**Figure 4:**
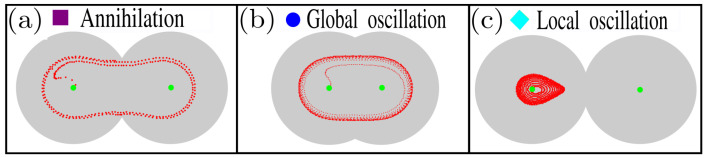
Snapshot of the trajectories for each regime in (**a**) the skyrmion annihilation, (**b**) global oscillation, and (**c**) local oscillation. These snapshots are obtained for the parameters (**a**) J=2.5 A/μm${}^{2},\;\beta =0.7$, (**b**) J=0.5 A/μm${}^{2},\;\beta =0.4$, and (**c**) J=0.01 A/μm${}^{2},\;\beta =0.9$. The red dot represents the position of the skyrmion, while the green dot represents the electrodes.

Figure 4 illustrates the trajectories for three different dynamics obtained in the SD. Figure 4a–c presents a typical annihilation state and global and local oscillatory regimes, respectively. In the Supplementary Material, videos of each state are shown.

#### 3.2.1. Annihilation Regime

**Figure 5:**
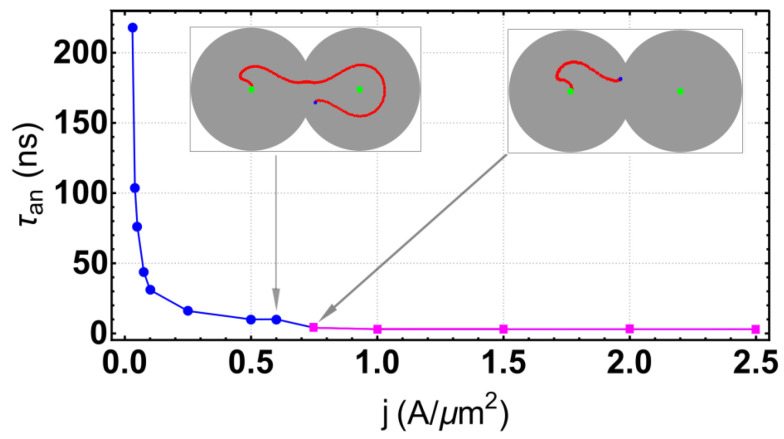
Lifetime of the skyrmion before annihilation as a function of current density *J* at β=0.9. The inserts illustrate the annihilation types.

Annihilation regimes are characterized by skyrmion annihilation after a transient time and the creation of a uniform magnetization in the two-disk system, except at the borders. This regime appears for high values of β and *J*, as shown in the SD. We highlight here that there are at least two different skyrmion trajectories before annihilation. In the first one, the skyrmion covers the entire system, moving around a well-defined orbit, and after a transient, the skyrmion annihilates at some part of the system. In the second annihilation scenario, the skyrmion is destroyed faster in the surrounding disk, where it starts its motion. The annihilation type depends on the current density. To emphasize this point, we fix the inter-disk separation parameter at β=0.9 and recollect the transient time as a function of the density current *J*, as presented in Figure 5. One can notice that the annihilation can be characterized by the skyrmion lifetime, which decreases with the current. We also observe that there is a value of $\tau_{an}^{*}\approx 0.75$ such that for $\tau_{an}<\tau_{an}^{*}$, the skyrmion trajectory occupies just the left region, whereas for $\tau_{an}>\tau_{an}^{*}$ the skyrmion trajectory occupies the entire system. In the first annihilation case, the lifetime follows a stretched exponential law, $\tau_{an}\left(J\right)=a\exp\left(b/J^{p}\right)$ [32], where (a,b,p)≈(2.399,0.179,0.522), which are commonly found in irreversible processes. Nevertheless, the lifetime remains almost constant in the second regime.

#### 3.2.2. Oscillatory Regimes

The oscillatory regimes are characterized by a stable and well-defined orbit. These regimes appear for small and intermediate values of β and *J*, as it is shown in the SD. Two types of oscillations are observed, which can be denoted as global and local, as illustrated in Figure 4. The local steady-state is represented by an orbit restricted to one region, in our case, the left region. The global regime considers a trajectory that covers all the systems.

**Figure 6:**
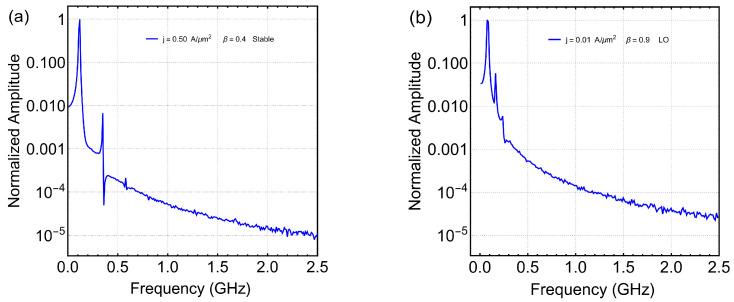
Fast Fourier Transform of the *z*-component of magnetization spatial average, $\langle M_{z}\rangle$, as a function of the frequency at $\left(J,\beta \right)=\left(0.5\;A/\mu m^{2},0.4\right)$ (**a**) and $\left(J,\beta \right)=\left(0.01\;A/\mu m^{2},0.9\right)$ (**b**). Here, panel (**a**) represents a global oscillation, while panel (**b**) represents a local oscillation.

The obtained results evidence that the times series of all magnetization components are regular and that they can be described by the Fast Fourier Transform (FFT). Figure 6 shows the FFT of the *z*-component of magnetization spatial average, $\langle M_{z}\rangle$, as a function of the frequency for two different sets of (J,β). Figure 6a depicts the corresponding FFT obtained for the global oscillatory regime, whereas Figure 6b is for the local one. We can observe that, in both cases, there are two relevant peaks in the spectrum, but in the global oscillation, the value of the second peak is smaller than in the local one, and the distance between the frequency’s values is larger for the global oscillation. This implies that the behavior is more regular for global oscillation.

**Figure 7:**
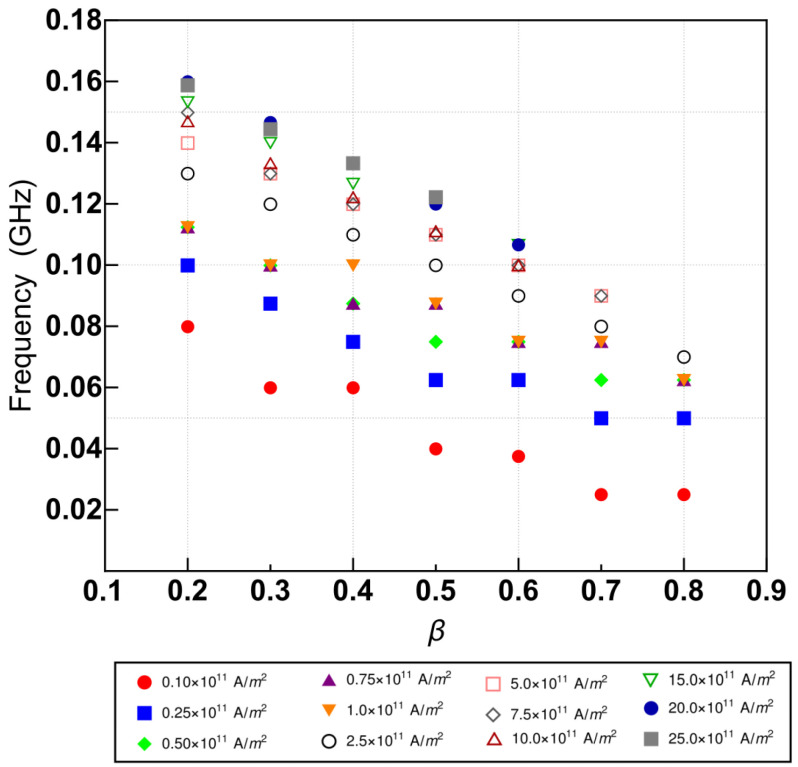
Value of frequency where the FFT is at its maximum as a function of β for different values of *J*.

To characterize the global oscillations, we have computed the corresponding Fast Fourier Transformation for each state in the regime. Figure 7 shows the maximum value of the frequency obtained from FFT as a function of β for different values of *J*. We observe that it decays almost linearly with β, which implies that the oscillation’s periods increase with β. Moreover, we can notice that the frequency increases with the density current with a ladder trend, implying a decrease in the oscillation period.

Finally, let us remark that the appearance of global oscillations is much more robust than the local one. One can change this situation if the initial conditions are modified. Nevertheless, this issue is out of the scope of this work, and it will be explored in future projects.

## 4. Conclusions

In this work, we presented an analysis of the dynamical behavior of one skyrmion in an STNO device with a double-disk geometry under the effect of an electric current. The obtained results allow us to construct a full 2D state diagram of the skyrmion’s dynamical behavior as a function of the degree of the disk’s connection and the electric current density. Three different regimes were obtained: (a) a skyrmion annihilation, (b) a global oscillatory state, and (c) a local oscillatory state. For the annihilation state, we found that the transient time decays within a stretched exponential law as a function of the electric current. For the global oscillatory states, the fundamental modes were obtained as a function of the system parameters, finding that the maximum frequency shows a linear dependence on the degree of connection between the disks. We expect that the presented state diagram motivates and guides experimental work in order to obtain these specific behaviors for new applications based on skyrmion dynamics. The system considered in this work allows the analysis of several effects on the static and dynamics of skyrmions in a double-disk system. For instance, this system could give place to a chaotic regime for the system hosting two or more skyrmions. Additionally, by considering a double-disk with thermal noise, a wide variety of phenomena can be observed, such as those reported for skyrmions displacing in nanomagnets having pinning potential [33]. 

## Data Availability

All data that support this study are included within the article (and any Supplementary File).

## Supplementary Materials

The following supporting information can be downloaded at: https://www.mdpi.com/article/10.3390/nano12183086/s1.

## Abbreviations

The following abbreviations are used in this manuscript:

DMI	Dzyaloshinskii–Moriya Interaction
STNO	Spin Torque Nano-Oscillator
LLGS	Landau–Lifshitz–Gilbert–Slonczewski
STT	Spin Transfer Torque
J	Electric current density
SD	State Diagram
FFT	Fast Fourier Transform
